# Childhood multisystem inflammatory syndrome associated with COVID-19 (MIS-C): a diagnostic and treatment guidance from the Rheumatology Study Group of the Italian Society of Pediatrics

**DOI:** 10.1186/s13052-021-00980-2

**Published:** 2021-02-08

**Authors:** Marco Cattalini, Andrea Taddio, Claudia Bracaglia, Rolando Cimaz, Sara Della Paolera, Giovanni Filocamo, Francesco La Torre, Bianca Lattanzi, Alessandra Marchesi, Gabriele Simonini, Gianvincenzo Zuccotti, Fiammetta Zunica, Alberto Villani, Angelo Ravelli, on behalf of the Rheumatology Study Group of the Italian Society of Pediatrics

**Affiliations:** 1 Pediatrics Clinic, University of Brescia and ASST Spedali Civili di Brescia, Piazzale Spedali Civili 1, 25123 Brescia, Italy; 2 University of Brescia, P.zza Del Mercato 15, Brescia, Italy; 3 Institute for Maternal and Child Health, IRCCS “Burlo Garofolo”, Via dell’Istria 65/1, 34137 Trieste, Italy; 4 University of Trieste, Piazzale Europa 2, 34100 Trieste, Italy; 5 Division of Rheumatology, Bambino Gesù Children’s Hospital, IRCCS, Piazza di Sant’Onofrio, 4, 00165 Rome, Italy; 6 Department of Clinical Sciences and Community Health, University of Milan, Via Commenda 19, 20122 Milan, Italy; 7 Pediatric Intermediate Care Unit, Fondazione IRCCS Ca’ Granda Ospedale Maggiore Policlinico, Via della Commenda 9, 20122 Milan, Italy; 8 Pediatric Rheumatology Center, Pediatric Unit, “Giovanni XXIII”, Pediatric Hospital, Via Giovanni Amendola 207, 70126 Bari, Italy; 9 SOD Pediatria, Ospedali Riuniti, Via Conca 71, Torrette, 60126 Ancona, Italy; 10 Bambino Gesu’ Children’s Hospital, IRCCS, Piazza Sant’Onofrio 4, 00165 Rome, Italy; 11 Pediatric Rheumatology Unit, AOU Meyer, University of Florence, Via Gaetano Pieraccini 24, 50139 Florence, Italy; 12 Department of Pediatrics, Children’s Hospital V Buzzi, University of Milan, Via Lodovico Castelvetro 32, 20154 Milan, Italy; 13 Clinica Pediatrica e Reumatologia, IRCCS Istituto Giannina Gaslini and DINOGMI, Università di Genova, Via Gerolamo Gaslini 5, 16147 Genoa, Italy

## Abstract

**Background:**

Italy was the first Western country to be hit by the SARS-CoV-2 epidemic. There is now mounting evidence that a minority of children infected with SARS-CoV2 may experience a severe multisystem inflammatory syndrome, called Multisystem inflammatory Syndrome associated with Coronavirus Disease 2019 (MIS-C). To date no universally agreed approach is available for this disease.

**Main body:**

as Italy is now facing a second hity of COVID-19 cases, we fear a recrudescence of MIS-C cases. We have, therefore, decided to prepare a report that will help clinicians to face this novel and challenging disease. We propose a diagnostic algorithm, to help case definition and guide work-up, and a therapeutic approach. MIS-C should be promptly recognized, based on the presence of systemic inflammation and specific organ involvement. Early treatment is crucial, and it will be based on the combined use of corticosteroids, high-dose immunoglobulins and anti-cytokine treatments, depending on the severity of the disease. Ancillary treatments (such as. aspirin and thrombo-profilaxis) will be also discussed.

**Conclusions:**

we propose a document that will help physicians to diagnose and treat MIS-C patients. Given the level of evidence available and the methodology used, this document should not be interpreted as a guideline; the final decision about the optimal management should still be taken by the caring physician, on an individual basis.

## Introduction

Italy was the first Western country to be hit by the SARS-CoV-2 epidemic. To date, more than 943,000 cases have been diagnosed, with more than 41,192 deaths. Children accounted for around 2% of infections, with an estimated mortality rate of 0,2% [1]. These figures confirm the previous observation in China that children develop milder forms of the illness, compared to adults [2–4]. Nonetheless, there is now mounting evidence that a minority of children infected with SARS-CoV2 may experience a severe multisystem inflammatory syndrome, which has been named Pediatric Multisystem inflammatory Syndrome temporally associated with COVID-19 (PIMS-TS) in the UK and Multisystem inflammatory Syndrome associated with Coronavirus Disease 2019 (MIS-C) in the US [5–7]. The latter term will be used in this paper. The clinical spectrum of MIS-C is wide, and children have been treated with a variable association of intravenous immunoglobulin (IV Ig), high-dose glucocorticoids, and anti-cytokine medications [8–13]. To date, although diagnostic and therapeutic recommendations have been proposed by various pediatric societies, no universally agreed approach is available [14, 15].

After the first epidemic peak, which began in late February, the national lockdown policy in Italy led to a drastic reduction of cases, that, however, have restarted growing in the recent weeks. As MIS-C cases have been mostly observed in the regions with the highest impact of SARS-CoV-2 infection, we fear a recrudescence of the disease throughout Italy. We have, therefore, decided to prepare a report that helps clinicians to face this novel and challenging disease. Given the limited information currently available and the methodology employed, this document should not be seen as a guideline, but simply as a set of clinical suggestions based on the existing literature and the personal experience of the authors.

## Case definition

There are multiple case definition criteria for MIS-C [16]. We propose to consider MIS-C diagnosis in the presence of:

A child or adolescent with

**Fever** (> 38 °C) lasting for more than 24 h.

**+**

Signs/symptoms of at least 2 **organs involvement**^**a**^

**+**

Laboratory work-up showing **systemic inflammation** (leukocytosis with neutrophilia, ESR and CRP (and PCT) increase, with or without **lymphopenia**

**+**

Exclusion of **infection**^**b**^

^b^ a recent exposure to SARS-CoV2 may be demonstrated in the majority of patients by means of nasal/pharyngeal swabs or serology. In case of high clinical suspicion, MIS-C diagnosis and treatment should not be delayed by a negative swab or serology. A personal history of close SARS-CoV contact is present in the majority of cases and may be sufficient to substantiate MIS-C hypothesis.

^a^**ORGAN INVOLVEMENT**

**Table:** 

**HEART** ^c^ in case of coronary dilation, we recommend to refer to related AHA definitions [17]	Hypotension. Please consider that some patients with MIS-C may have SHOCK as the presenting sign, or develop it rapidly during hospitalization. This shock is usually associated with capillary leak syndrome or is cardiogenic, without signs of hypoperfusion. Myocarditis (in some cases there is only cardiac enzyme elevation, without ultrasound abnormalities) Valvular insufficiency Cardiac conduction abnormalities Heart failure Coronary abnormalities^c^
**Respiratory**	Nasal drip/congestion Pharyngodynia/pharyngitis Cough Thoracic pain Respiratory distress Acute respiratory failure
**Skin and mucous membranes**	Polymorphous rash/perineal erythema Erythema of the palms and soles /induration of the hands and feets Cracked lips/strawberry tongue Nonexudative conjunctival injection Lymphnode enlargement
**Kidney**	Renal failure Oliguria and/or anuria Oedema
**Gastrointestinal**	Severe abdominal pain Diarrhea Nausea and/or vomiting Jaundice
**Musculoskeletal**	Arthralgia Myalgia Arthritis
**Central nervous system (CNS)**	Headache Irritability Meningism Confusion Seizures

**Fig. 1:**
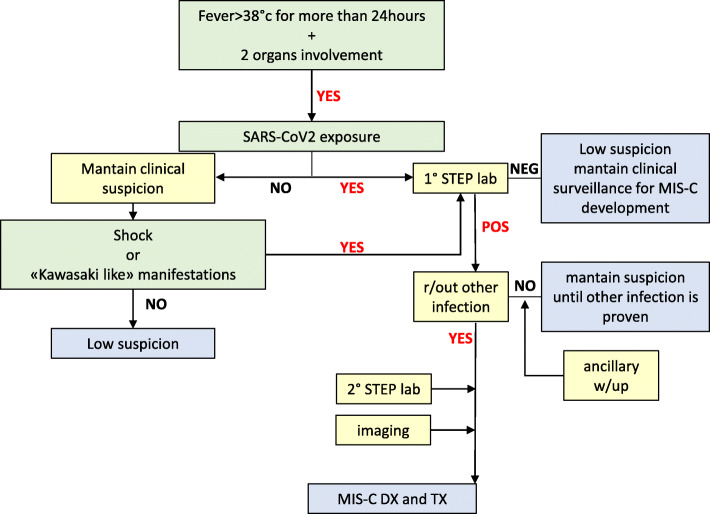
A proposed diagnostic algorithm for children with suspected MIS-C. Please refer to the main text for further details

As many of the signs and symptoms listed are not specific, MIS-C diagnosis should rely on a high index of suspicion and cautious clinical judgement, taking into account the patient’s history, the severity of organ involvement, the inflammatory markers level and other possible mimickers (Fig. 1).

**LABORATORY WORK-UP**

**Table:** 

**First Step** All of the following labworks should be performed in all suspected MIS-C as soon as possible *See Ravelli et al. and Henter et al for criteria [18, 19]	**Complete blood count**: leukocytosis with lymphopenia is typical. In case of leukopenia, thrombocytopenia or anemia, consider sHLH* **CRP**: CRP elevation is typical **Coagulation**: Hyperfibrinogenemia is typical, PT and PTT should be obtained to investigate a prothrombotic state. In case of low fibrinogen, consider sHLH*. In case D-dimer is measured, high levels should be interpreted as potentially related to the hyperinflammatory state. **Electrolytes**: hyponatremia may occur. **Liver function tests**: in case of abnormal liver function tests, consider sHLH*. MIS-C cases with gallbladder hydrops (that may cause hyperbilirubinemia) have been described. **Kidney function tests**: MIS-C cases with acute kidney injury have been described. **Blood gas analysis**: to assess gas exchange and the presence of metabolic acidosis. High lactates have been described in MIS-C patients without evidence of sepsis
**Second Step** Should be performed in case hyperinflammation is confirmed by first step laboratory test, and in the presence of at least one typical clinical finding	**Peripheral smear**: to look for schistocytes or Burr cells, denoting microangiopathy **Acute phase reactancts**: high level of pro-calcitonin has been described in patients with MIS-C; in case of very high ferritin levels (with ESR fall and high CRP) consider sHLH* **Troponins** and **NTpro-BNP**: to rule out myocarditis, which is a very common finding. Troponin and NT pro-BNP should be first step labworks in case myocarditis is suspected **Total protein** and **albumin** levels: hypoalbuminemia may occur **Triglycerides**: consider sHLH* in case of hypertriglyceridemia **CPK**, **LDH**: may indicate myopathy or cytolysis **C3**, **C4**: complement consumption may be seen **γGT**: together with LFTs may denote liver involvement **Amylase**, **lipase**: pancreatitis may occur
**Ancillary tests** As the main differential diagnosis is with sepsis, all possible tests to rule out infection should be performed, according to clinical suspicion. These may include (but should not be limited to) the following **N.B.** MIS-C cases with (presumed) co-infection by EBV, *Mycoplasma Pneumoniae, Staphylococcus aureus* have been described. A positive test for infection should not exclude MIS-C diagnosis in case of high suspicion	**Blood, urine, stool cultures** **Serologies** for: *EBV, Mycoplasma Pneumoniae, Coxackievirus, Echovirus, Adenovirus, Influenza, VRS*. In case of positive serologies, **PCR** testing should be obtained, whenever possible Naso-pharyngeal **swabs** for viruses

**IMAGING**

**Table:** 

To be performed in case of suggestive clinical findings and first step consistent with hyperinflammation	**Chest X-Ray**: the most common finding is interstitial pneumonia. Pleurisy or heart shadow enlargement may be present **EKG + Echo-Cardiogram**: to seek for signs of myocarditis (if cardiac enzymes are increased or in case of clinical suspicion), valvular insufficiency, pericarditis, cardiac tamponade, coronary abnormalities. In case of shock, echo-cardiogram may be helpful to rule out dehydration **Abdomen US**: in case of gastrointestinal symptoms. Possible findings are: hepato/splenomegaly, peritoneal fluid, hepato/splenomegaly **Chest CT**: if indicated by clinical picture and X-ray results **Heart MRI**: if indicated by clinical picture and ecocardiogram results **Colonscopy**: in case of severe gut disease

## Treatment

To date, there is limited evidence to establish the optimal therapeutic approach to a child with MIS-C. Given the partial overlap of the clinical manifestations of MIS-C with those of Kawasaki disease, the majority of patients have been treated with the standard therapeutic protocols for the latter illness [17]. It is important to consider that the spectrum of clinical manifestations and severity of MIS-C is is wide. Thus, the best treatment approach should be defined on an individual basis, and the following proposals are to be interpreted only as suggestions.

**Table:** 

**Intravenous immunoglobulin**	2 g/kg IV (up to 70-80 g) to be administered over at least 12 h. In patients with heart failure immunoglobulins should be administered over at least 16 h or, alternatively, the total dose should be splitted in two infusions 12 h apart. A second dose of immunoglobulins should be considered in case of inadequate response
**Glucocorticoids** To be administered with IVIg upfront in case of heart involvement, severe disease, impending sHLH or toxic shock syndrome. i or ii should be chosen depending on disease severity, based on clinical/laboratory features. Metylprednisolone pulses are recommended in case of sHLH diagnosis/suspicion	i. Methylprednisolone 1 mg/kg BID IV ii. Metylprednisolone 30 mg/kg (max 1 g) IV pulse q1d for 1–3 days, followed by Metylprednisolone i.v./Prednisone orally, based on the severity of clinical/laboratory features iii. Consider Dexamethasone 10 mg/m^2^ q1d in case of sHLH or CNS involvement
**Biologic medications** i. to be used SQ as second line treatment, in case of persistent disease activity 48 h after first-line treatment or in case of sHLH. ii.-iii. to be used IV in adjunction to corticosteroids and IVIg in case of severe sHLH or shock with cardiac failure	i. Anakinra: 4-6 mg/kg q1d SQ ii. Anakinra: 2 mg/kg IV (max 100 mg/dose) × 4/day (to be diluted in 100 sterile saline and administered in no more than 1 h) iii. Anakinra: 2 mg/kg (max 100 mg) IV. pulse followed by continuous infusion at a total daily dose of no more than 12 mg/kg or 400 mg
**Ancillary treatments**	Large-spectrum antibiotics: while waiting for microbiology tests Acetylsalicilic acid: 5 mg/kg for at least 6–8 wks. In case coronary abnormalities are found, refer to AHA recommendations for Kawasaki Disease [17] Proton Pump Inhibitor: as needed Thromboprophylaxis with LMWH: since adults with COVID-19 are at high risk of thromboembolism, and given the high inflammatory state of children with MIS-C, it appears reasonable to start prophylaxis with LMWH. As per ISTH recommendations [20], risk stratification should be done based on D-Dimer and other known pro-thrombotic factors. In case of D-Dimer >5X normal values and/or presence of other known pro-thrombotic factors, Enoxaparin 100 UI/kg BID should be administered. Eculizumab: in case of acute kidney failure and evidence of microangiopathy, consider treatment with eculizumab [21]

Since MIS-C is a post-infectious disease, it is conceivable to assume that symptoms have their onset when the viremic phase is ended. Nonetheless, it is difficult to clearly differentiate these two phases (viremic vs hyperinflammatory) in some clinical scenarios. We recommend to consider carefully the appropriate timing to start immunomodulatory treatment in such cases, to avoid interference with anti-viral host response.

## Conclusions

Since there is a resurgence of COVID-19 cases throughout Italy, we expect a rise in MIS-C patients over the next weeks. Although MIS-C has variable severity, the majority of patients are seriously ill. The clinical experience indicates that prompt recognition and timely treatment are crucial to achieve good outcomes. Given the frequent overlap of clinical manifestations between MIS-C and Kawasaki disease, patients with the hyperinflammatory syndrome have generally been treated with the therapeutic protocols used in Kawasaki disease. Since the available information does not allow to formulate well-established guidelines or recommendations for MIS-C treatment, and the long-term sequelae of the illness are not yet known, we agree with the therapeutic regimens proposed and adopted so far. The final decision about the optimal management should be taken by the caring physician, based on the disease characteristics and severity of each individual patient.

## Data Availability

not applicable.

## Abbreviations

SARS-CoV2: Severe Acute Respiratory Syndrome – CoronaVirus 2
COVID-19: Coronavirus Disease 2019
PIMS-TS: Pediatric Multisystem inflammatory Syndrome temporally associated with COVID-19
MIS-C: Multisystem inflammatory Syndrome associated with Coronavirus Disease 2019
